# Phosphorus Restriction in Brooding Stage Has Continuous Effects on Growth Performance and Early Laying Performance of Layers

**DOI:** 10.3390/ani11123546

**Published:** 2021-12-14

**Authors:** Lan Li, Xiaoyi Zhang, Lihong Zhao, Jianyun Zhang, Cheng Ji, Qiugang Ma

**Affiliations:** State Key Laboratory of Animal Nutrition, College of Animal Science and Technology, China Agricultural University, Beijing 100193, China; lanli0928@163.com (L.L.); zxyxjljy@163.com (X.Z.); zhaolihongcau@cau.edu.cn (L.Z.); jyzhang@cau.edu.cn (J.Z.); jicheng@cau.edu.cn (C.J.)

**Keywords:** layer, phosphorus, brooding period, growth performance, laying performance

## Abstract

**Simple Summary:**

Phosphorus plays a critical role in bone and eggshell formation. Dietary phosphorus oversupply depletes non-renewable natural resources and causes environmental concerns in animal husbandry. This study evaluated the effects of phosphorus restriction in the brooding stage and subsequent recovery on growth performance, tibia development and early laying performance of layers. Phosphorus restriction decreases growth performance and bone characters in the brooding stage, and the adverse effects on body weight and early laying performance do not disappear after phosphorus supplementation. These findings give a foundation and new perspective on low phosphorus feeding strategies in the production of layers.

**Abstract:**

This study evaluated the effects of phosphorus restriction in the brooding stage and subsequent recovery on growth performance, tibia development and early laying performance of layers. 360 one-day-old hens were randomly divided into 4 groups with 6 replicates and 15 chicks per replicate. Chicks were fed diets containing 0.13% (L), 0.29% (M), 0.45% (N), 0.59% (H) non-phytate phosphorus (nPP) from 1 to 8 weeks of age. From 9 to 20 weeks of age, the L and N group were divided into two groups fed normal level phosphorus (n, 0.39% nPP) and high-level phosphorus (h, 0.45% nPP) separately, then all the birds were fed a normal diet (0.39% nPP) from 21 to 26 weeks of age. Four treatments were tested: Ln, Lh, Nn, and Nh. The lower body weight, average daily feed intake, tibia length and daily tibial increment were observed in the L group (*p* < 0.05) and the ratio of feed to gain was significantly increased in the L group at 8 weeks of age (*p* < 0.05). In addition, the fresh and degreased tibia weight, bone ash, Ca content in the tibia and P content in the ash and tibia were significantly decreased in the L group at 8 weeks of age (*p* < 0.05). After compensatory processes, there was no significant difference in tibia characters; however, body weight in the Ln group was significantly lower than in the Nn group (*p* < 0.05) and was significantly lower in the Lh group than the Nn group (*p* < 0.01) and Nh group (*p* < 0.05). In addition, the laying rate and average daily egg mass in the Lh group were lower than Nn and Nh (*p* < 0.05). In conclusion, severe dietary phosphorus restriction impaired growth performance and bone mineralization in the brooding stage. Subsequent phosphorus supplementation could not alleviate this adverse effect on body weight, which continued to affect egg production. These findings give a foundation and new perspective on a low phosphorus feeding strategy in layer production.

## 1. Introduction

Phosphorus is an essential mineral nutrient for animals, which not only plays a vital role in metabolism, but is also a necessary component of nucleic acids, phospholipids, adenosine triphosphate (ATP), several coenzymes and bone [1]. Due to the amount of available phosphorus provided by plant-based feed ingredients being regarded as insufficient, diets for poultry are usually supplemented with mineral feed phosphates or microbial phytase [2]. However, feed phosphates from mined rock phosphate are non-renewable; thus, a decrease in the use of feed phosphates phosphorus is required to make egg production more sustainable [3,4]. In addition, over-supplementation of phosphorus also leads to increased feed costs and a higher concentration of phosphate in the manure [5]. This can contribute to environmental problems, such as the eutrophication of sensitive ecosystems [6,7,8].

The adaptation of animals to low phosphorus diets has been recognized previously. Yan et al. demonstrated that broiler fed nPP-deficient diets during an early phase of growth and later fed a diet with adequate minerals had higher phosphorus utilization and bone mineralization [9]. A recent study has found that a reduction in dietary nPP levels (up to 0.15 percent) had no adverse effects on health and laying performance, structure or quality of bone and the P transporters related gene expression; however, it significantly lowered phosphorus excretion in laying hens [10]. However, another study reveals that low phosphorus diets significantly influence the laying hens, decreasing the laying performance and egg quality, causing damage to the microstructure and reducing the biomechanical properties of the tibia [11]. Despite increasing scientific reports on the application of low phosphorous in animal husbandry, the continuous effect of phosphorus restriction in the brooding stage on subsequent development and reproduction performance, especially in layers, is still not clear. As the starting period in laying hens’ production, the growth and development of chickens at the brooding stage are directly related to growth performance during the rearing period and the production performance of the laying period. Therefore, we hypothesized that low phosphorous supplementation at the brooding period might affect growth performance and continuously affect laying performance. The purpose of this study was conducted to investigate the effects of phosphorus restriction in the brooding stage and subsequent recovery on growth performance, tibia development and early laying performance of laying hens.

## 2. Materials and Methods

The animal experimental and sample collection were approved by the Animal Care and Use Committee of China Agricultural University (AW13301202-1-14). All procedures were performed strictly following the Guide for Experimental Animals (Beijing, China) in this study.

### 2.1. Birds, Diets and Housing

A total of 360 1-day-old hens (Jingfen layer strain with a light weight, white feather and pink eggshell, cultivated by Huadu Yukou company, Beijing, China) with average body weight (BW) of 37.55 ± 0.30 g was randomly allotted into 4 groups with 6 replicates of 15 chicks per replicate. From 1 to 8 weeks: This stage used a completely single factor randomized design with four dietary non-phytate phosphorus (nPP) levels: low-level (L, nPP 0.13%), mid-level (M, nPP 0.29%), normal-level (N, nPP 0.45%) and high-level (H, nPP 0.59%). According to the broken line regression of growth performance (Table S1), 0.45% nPP was sufficient to meet the nutrient requirement for layers in 1 to 8 weeks of age. In addition, there was no significant difference in growth performance between N and H groups. Therefore, the N group was used as the control group in the subsequent recovery trial. From 9 to 20 weeks: a completely randomized design with two brooding period phosphorus levels (L and N) and two later-period phosphorus levels (n, 0.39% nPP and h, 0.45% nPP) factorial arrangement. Thus, there were a total of four treatments (Ln, Lh, Nn, Nh) in this stage. From 21 to 26 weeks: All the birds were fed a normal commercial diet (nPP, 0.39%).

The chicks were allowed ad libitum access to diets and water. A 23-h lighting regime was carried out during the first 3 days, 22 h of lighting with 2 h of darkness was used during 4~7 days, and then an increase of 2 h of darkness per week from 8 days to 49 days. Laying hens were maintained on a 9 h light schedule from 8 weeks of age to 17 weeks of age. Then, increasing 1 h of illumination each week from 18 weeks of age until reaching 16 h. During the first week, the temperature of the animal chamber was maintained at around 35 °C and then gradually lowered to get a constant temperature of 25 °C for the rest of the trial. The immune procedure was carried out in accordance with the breeding manual of layers. The corn-soybean meal diets (Table 1 and Table 2) were designed to meet the nutrient requirements of the “Chicken Feeding Standard” (NY/T33-2004), except phosphorus.

### 2.2. Growth Performance

At the end of 8, 10, 12, 16, and 20 weeks of age, collective live weight, residual feed weight of each replicate and tibia length were recorded. The BW, average daily feed intake (ADFI), average daily gain (ADG), the ratio of feed to gain (F/G) and daily tibial increment were calculated.

### 2.3. Tibia Characteristics

Tibias were collected from the left legs to determine fresh and degreased tibia weight, bone ash and calcium and phosphorus content in ash. The fresh weight of the left tibia was measured. The tibias were defatted by soaking in petroleum ether for 96 h, and then the tibias were dried at 105 °C for 24 h before the degreased tibias were weighted. After that, the degreased tibias were ashed for 12 h in a muffle furnace at 550 °C, and the ash weight was recorded. Calcium and phosphorus contents in ash were analyzed using a spectrophotometer [12]. The contents of calcium and P in the tibia were calculated.

### 2.4. Laying Performance

All eggs were gathered by hand at 15:00 every day, the total number of eggs and total egg weight in each replicate were recorded from the first day that laying started to the end of 26 weeks of age. The average egg weight, daily egg mass and laying rate were calculated.

### 2.5. Statistical Analyses

The data were provided as mean with standard error of the mean (SEM) and analyzed statistically by a general linear model (GLM) and broken line regression using SAS 9.4. Regression estimation was conducted to estimate hens’ linear and quadratic responses to different levels of nPP during the brooding period. Then the data during growing and laying periods were analyzed as 2 × 2 factorial with two brooding period phosphorus levels (L and N) and two later-period phosphorus levels (n and h). After one-way analysis of variance, Duncan’s multiple range tests were applied for specific differences. When the *p* value was less than 0.05, differences were considered statistically significant.

## 3. Results

### 3.1. Growth Performance

**Figure 1:**
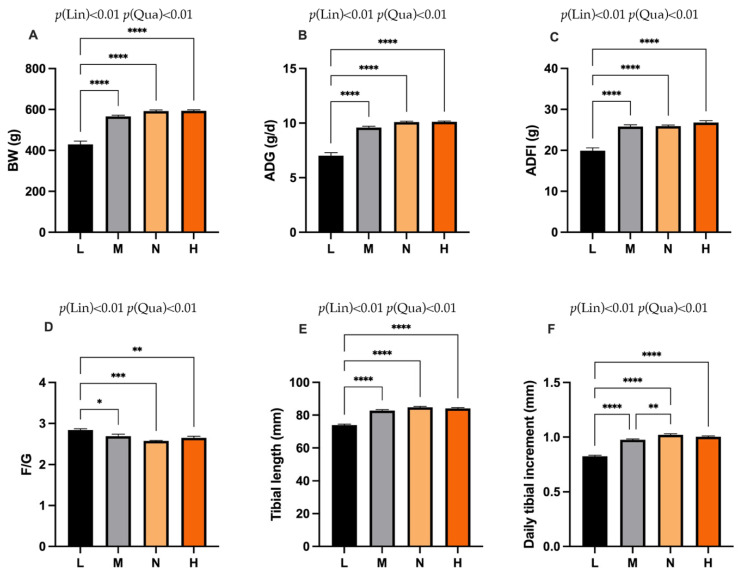
Effects of phosphorus restriction on growth performance of layers at 8 weeks of age. (**A**) BW (**B**) ADG (**C**) ADFI (**D**) F/G (**E**) Tibial length (**F**) Daily tibial increment. BW = body weight, ADG = average daily gain; ADFI = average daily feed intake; F/G = the ratio of feed to gain. Data are shown as mean ± SEM. * *p* < 0.05, ** *p* < 0.01, *** *p* < 0.001, **** *p* < 0.0001, P(Lin) = *p*-value (linear effect), *p* (Qua) = *p*-value (quadratic effect). L = group fed 0.13% nPP; M = group contained 0.29% nPP; N = group contained 0.45% nPP; H = group contained 0.59% nPP. *n* = 6 per group.

As shown in Figure 1, the results showed that the BW, ADFI, ADG, tibia length and daily tibial increment in the L group were significantly lower compared with other groups (*p* < 0.0001). However, the F/G in the L group was significantly higher than the M group (*p* < 0.05), N group (*p* < 0.001) and H group (*p* < 0.01). Moreover, the daily tibial increment in the M group was significantly reduced compared with the N group (*p* < 0.01).

**Figure 2:**
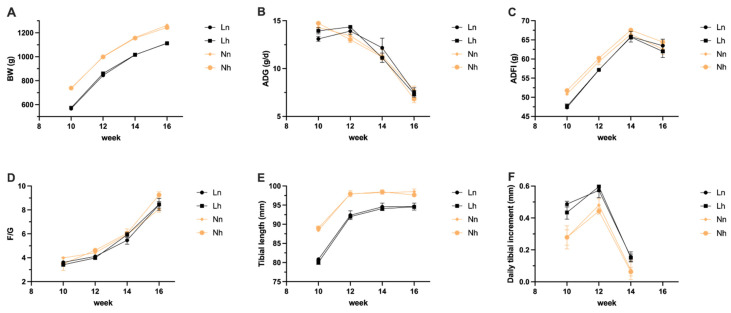
Effects of phosphorus restriction in brooding stage and subsequent recovery on growth performance of layers during 10–16 weeks of age. (**A**) BW (**B**) ADG (**C**) ADFI (**D**) F/G (**E**) Tibial length (**F**) Daily tibial increment. BW = body weight, ADG = average daily gain; ADFI = average daily feed intake; F/G = the ratio of feed to gain. Ln = group fed 0.13% nPP at 1–8 weeks and fed 0.39% nPP at 9–20 weeks; Lh = group fed 0.13% nPP at 1–8 weeks and fed 0.45% nPP at 9–20 weeks; Nn = group fed 0.45% nPP at 1–8 weeks and fed 0.39% nPP at 9–20 weeks; Nh = group fed 0.45% nPP at 1–8 weeks and fed 0.45% nPP at 9–20 weeks. Data are shown as mean ± SEM.

As shown in Figure 2, although the trend of ADG in Ln and Lh group at 12 weeks of age was shown to be higher than the two N groups, the standardized mean of BW in both L groups was still lower. No significant difference in ADFI and F/G among the four groups was found (*p* > 0.05). In addition, the daily tibial increment was higher in two L groups; however, the tibia length was still lower than the two N groups at 16 weeks of age. In addition, there was no significant interaction between the nPP levels of the brooding period and those of later periods (*p* > 0.05).

**Figure 3:**
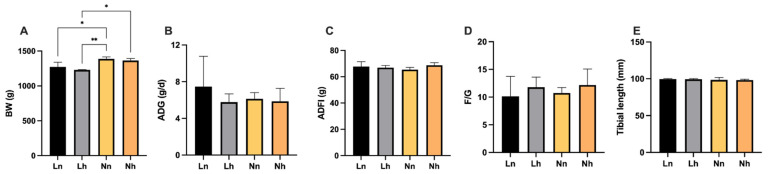
Effects of phosphorus restriction in brooding stage and subsequent recovery on growth performance of layers at 20 weeks of age. (A) BW (**B**) ADG (**C**) ADFI (**D**) F/G (**E**) Tibial length. BW = body weight, ADG = average daily gain; ADFI = average daily feed intake; F/G = the ratio of feed to gain. Data are shown as mean ± SEM. * *p* < 0.05, ** *p* < 0.01. Ln = group fed 0.13% nPP at 1–8 weeks and fed 0.39% nPP at 9–20 weeks; Lh = group fed 0.13% nPP at 1–8 weeks and fed 0.45% nPP at 9–20 weeks; Nn = group fed 0.45% nPP at 1–8 weeks and fed 0.39% nPP at 9–20 weeks; Nh = group fed 0.45% nPP at 1–8 weeks and fed 0.45% nPP at 9–20 weeks. *n* = 6 per group.

As can be seen from Figure 3, no significant differences in the ADG, ADFI and F/G could be found (*p* > 0.05) at 20 weeks of age. However, BW in Ln was significantly lower than the Nn group (*p* < 0.05), while BW the in Lh group was significantly lower than the Nn group (*p* < 0.01) and the Nh group (*p* < 0.05). There was no significant interaction between the nPP levels of the brooding period and those of later periods (*p* > 0.05).

### 3.2. Tibia Characters

**Figure 4:**
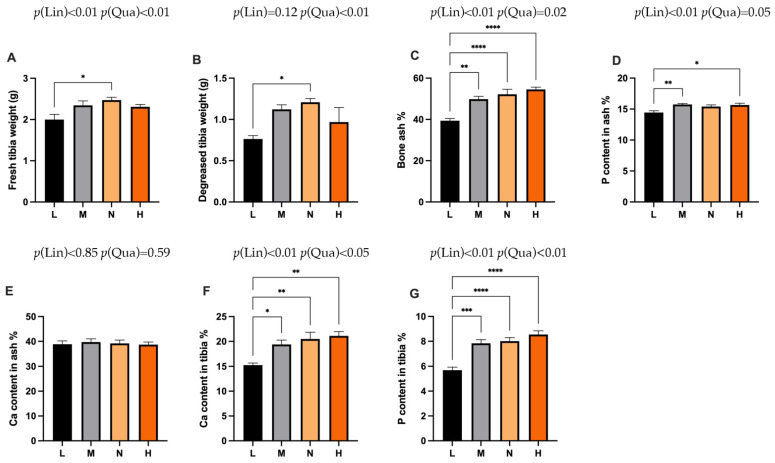
Effects of phosphorus restriction in brooding stage on tibia characters of layers at 6 weeks of age. (**A**) Fresh tibia weight (**B**) Decreased tibia weight (**C**) Bone ash (**D**) P content in ash (**E**) Ca content in ash (**F**) Ca content in tibia (**G**) P content in tibia. Data are shown as mean ± SEM. * *p* < 0.05, ** *p* < 0.01, *** *p* < 0.001, **** *p* < 0.0001, *p* (Lin) = *p*-value (linear effect), *p* (Qua) = *p*-value (quadratic effect). L = group fed 0.13% nPP; M = group contained 0.29% nPP; N = group contained 0.45% nPP; H = group contained 0.59% nPP. *n* = 6 per group.

In the brooding period, as shown in Figure 4, the fresh tibia weight and degreased tibia weight of the L group were significantly lower than the N group (*p* < 0.05). In addition, the tibia ash content was significantly lower than the M group (*p* < 0.01), N group (*p* < 0.001) and H group (*p* < 0.001). Moreover, the tibia calcium and phosphorus contents in the L group were significantly lower than the M group (*p* < 0.05), N group (*p* < 0.01) and H group (*p* < 0.01). The phosphorous content in ash in the L group was significantly decreased compared with the M group (*p* < 0.01) and H group (*p* < 0.05), while no significant differences in the ash calcium could be observed among the treatments.

**Figure 5:**
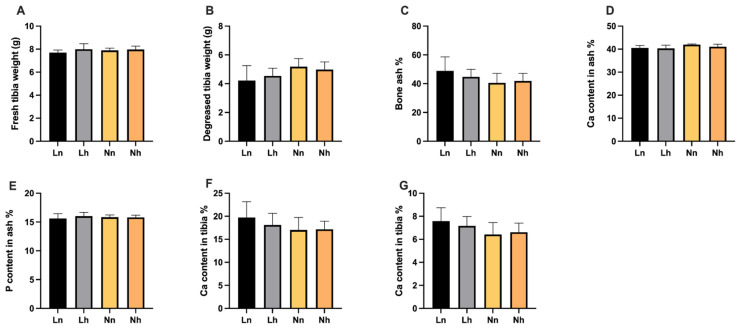
Effects of phosphorus restriction in brooding stage and subsequent recovery on tibia characters of layers at 20 weeks of age. (**A**) Fresh tibia weight (**B**) Decreased tibia weight (**C**) Bone ash (**D**) P content in ash (**E**) Ca content in ash (**F**) Ca content in tibia (**G**) P content in tibia. Data are shown as mean ± SEM. Ln = group fed 0.13% nPP at 1–8 weeks and fed 0.39% nPP at 9–20 weeks; Lh = group fed 0.13% nPP at 1–8 weeks and fed 0.45% nPP at 9–20 weeks; Nn = group fed 0.45% nPP at 1–8 weeks and fed 0.39% nPP at 9–20 weeks; Nh = group fed 0.45% nPP at 1–8 weeks and fed 0.45% nPP at 9–20 weeks. *n* = 6 per group.

Interestingly, in the later period (at 16 weeks of age), as can be seen from Figure 5, there were no significant differences in tibia characters were found among the treatments (*p* > 0.05). In addition, there was no significant interaction between the nPP levels of the brooding period and those of later periods (*p* > 0.05).

### 3.3. Early Laying Performance

**Figure 6:**
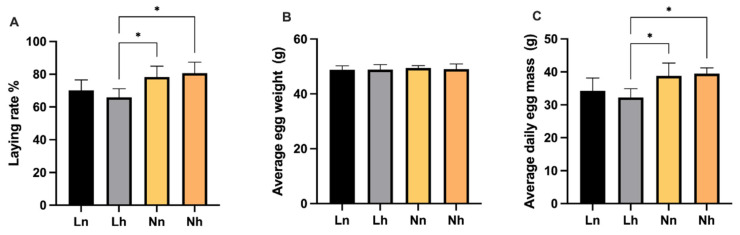
Effects of phosphorus restriction in brooding stage and subsequent recovery on laying performance of layers at 26 weeks of age. (**A**) Laying rate (**B**) Average egg weight (**C**) Average daily egg mass. Data are shown as mean ± SEM. * *p* < 0.05. Ln = group fed 0.13% nPP at 1–8 weeks and fed 0.39% nPP at 9–20 weeks; Lh = group fed 0.13% nPP at 1–8 weeks and fed 0.45% nPP at 9–20 weeks; Nn = group fed 0.45% nPP at 1–8 weeks and fed 0.39% nPP at 9–20 weeks; Nh = group fed 0.45% nPP at 1–8 weeks and fed 0.45% nPP at 9–20 weeks. *n* = 6 per group.

The laying performance results are provided in Figure 6. The laying rate and average daily egg mass in the Lh group were lower than Nn (*p* < 0.05) and Nh (*p* < 0.05). No significant differences in average egg weight were observed among the treatments (*p* >0.05). Moreover, there was no significant interaction between the nPP levels of the brooding period and those of later periods (*p* > 0.05).

## 4. Discussion

The brooding stage is crucial for the growth and development of the whole life cycle of laying hens [13]. Phosphorus is an essential mineral in poultry production because of its expressive participation in the eggshell quality and the bone’s metabolic and structural functions [14]. Therefore, it is of great significance to provide appropriate phosphorus during the brooding period of hens. Our study reveals that the adverse effects of dietary phosphorus restriction (0.13% nPP) were evident on growth performance during the brooding period. Phosphorus restriction (0.13% nPP) significantly decreased the BW, ADFI, ADG and increased F/G in 8 weeks of age. These findings are consistent with the results of Yan et al., who found that the broilers fed the low phosphorus diets weighed less than those fed the control diet at 18 days of age [9]. In addition, the low phosphorus diet significantly reduced the tibia length and tibia increment of hens, which is similar to the previous study [15], demonstrating that the low-phosphorus diet harms bone growth. Interestingly, there was no significant difference in growth performance in the M group. Mild decrease in P level (0.29% nPP) did not affect growth performance, but severe P deficiency (0.13% nPP) decreased the growth performance and bone increment in layers.

Studies have shown that ADG is a sensitive indicator that reflects the utilization of phosphorus [16,17]. A meta-analysis showed significant linear and quadratic effects of nPP on all responses, viz. ADG, feed intake, and feed efficiency in growing pigs [18]. In broilers, Valable et al. [19] confirmed that reducing dietary Ca and nPP in the grower phase does not impair the final growth performance, as long as the finisher diet provides enough Ca and nPP. In the present study, at 8–20 weeks of age, there was no significant difference between L and N groups in ADG, ADFI, F/G, and tibial length; however, the BW in the L group was still lower than the N group. This indicates that phosphorus repletion in a later period can compensate growth performance to some extent; however, severe P deficiency (0.13% nPP) will continuously affect BW until 20 weeks of age.

The brooding period is essential for the rapid bone development of laying hens [20]. Phosphorus is needed for laying hens, mostly for skeletal integrity [21]. Phosphate is necessary for bone formation and mineralization; rickets and osteomalacia will develop in its absence [22]. Tibia length is an effective indicator to evaluate the bone size of poultry [23]. Dietary calcium or phosphorus deficiency inhibits bone development in broilers via changing calcium or phosphorus metabolism-related characteristics in serum from day 1 to day 21 [24]. Another study has demonstrated that low dietary phosphorus induced thinning and fracturing of the bone trabeculae, as well as an increase of the bone marrow cavity of tibias [11]. Rousseau et al. [25] report that phosphorus deficiency in birds fed 0.30% NPP diets decreased bone growth and mineralization due to the lack of P for hydroxyapatite deposition. The contents of Ca and P and tibia ash increased curvilinearly with increasing dietary-available phosphorus [26]. In the current study, reduced dietary phosphorus levels (0.13% nPP) led to a decrease in fresh and degreased tibia weight, percentage of ash and Ca, and P contents in the tibia and ash at 6 weeks of age. This study confirms that nPP is associated with tibia characters in the brooding stage of layers.

The birds fed diets with P deficiency in the brooding stage and repletion in the later period had no significant difference in tibia characters. The disappearance of the adverse effects of P deficiency on growth performance in the later period may be due to the birds’ adaptability to P restriction. Research has shown that broilers exhibit high adaptability when exposed to P or Ca restrictions at an early stage [9]. Weanling pigs adapt to mild dietary P limitations via enhancing the absorption efficiency of phosphate by stimulating Na^+^-dependent phosphate uptake in the small intestine [27]. In addition, Rousseau et al. [25] studied chicks fed low-phosphorus diets for 10 days and found that, as dietary depletion increased, nPP relative transfer increased, implying an increase in the apparent digestibility of phosphorus. This indicates that laying hens fed with phosphorus restriction diets in the starter period suffer adverse effects on bone health; thus, increasing the phosphorus level in the later stage could compensate for the phosphorus deficiency, while alleviating the harm of early phosphorus deficiency. Therefore, although decreased bone mineralization occurred as a result of early-stage phosphorus deficiency, the deficit can be alleviated if the dietary phosphorus is replenished later.

The development of hens during the brooding stage reflects their reproductive performance. BW of hens at the beginning of laying production is a significant factor influencing laying performance; heavier hens produce larger eggs than lighter hens [28,29]. Therefore, in the brooding stage, efforts should be made to ensure the healthy weight of the hens, which will lay the foundation for the high production of laying hens in the future to obtain the best egg production effect and the best economic benefit. Nie et al. found that dietary nPP significantly affected laying performance; the average egg weights were quadratically correlated with levels of dietary nPP [30]. With no supplemental phosphorus or phytase, hens consuming the 0.10% available phosphorus diet had significantly lower egg production, feed intake and egg yield [31]. In this current study, our results indicated that the laying rate and average daily egg mass in the L group were significantly lower than the control group. These results could indicate that the early phosphorus deficiency would affect the laying rate, and increasing phosphorus level in the later stage could not improve the laying rate in the phosphorus deficiency group, which may relate to the decreasing BW of layers. Further results are needed to determine the mechanism of early phosphorus deficiency on laying rate.

## 5. Conclusions

In conclusion, severe phosphorus deficiency (0.13% nPP) decreases growth performance and bone growth in the brooding stage. After phosphorus supplementation, the adverse effects of bone can be alleviated; however, there are continuous effects on body weight. In addition, low phosphorous supplementation in the brooding stage harms laying performance. These findings have significant implications for understanding the effects of a low phosphorus diet in the early stage, as well as the continuous effect on growth and laying performance. Further experiments are needed to focus on early phosphorus restriction in laying performance and egg quality of laying hens during the whole egg production period to design feeding strategies that optimize P utilization.

## Figures and Tables

**Table 1:** Composition and nutrient levels of brooding period diets (%, air-drying basis).

Items	Treatment Groups ^3^			
	L	M	N	H
Ingredients (%)				
Corn	65.20	65.20	65.20	65.20
Soybean meal	29.40	29.40	29.40	29.40
Dicalcium phosphate	0.00	0.95	1.90	2.85
Zeolite powder	2.01	1.59	1.16	0.74
Limestone	2.35	1.82	1.30	0.77
Salt	0.30	0.30	0.30	0.30
Choline chloride (50%)	0.10	0.10	0.10	0.10
L-Lysine HCl (98%)	0.11	0.11	0.11	0.11
DL-methionine	0.19	0.19	0.19	0.19
Premix ^1^	0.34	0.34	0.34	0.34
Total	100.00	100.00	100.00	100.00
Nutrient levels ^2^				
Metabolizable energy (MJ/kg)	11.72	11.72	11.72	11.72
Crude protein	18.00	18.00	18.00	18.00
Utilizable methionine	0.45	0.45	0.45	0.45
Utilizable methionine and cystine	0.74	0.74	0.74	0.74
Utilizable Lysine	1.00	1.00	1.00	1.00
Utilizable Tryptophan	0.20	0.20	0.20	0.20
Utilizable Threonine	0.68	0.68	0.68	0.68
Calcium	0.98	0.96	1.02	1.06
Total phosphorus	0.41	0.52	0.67	0.86
Non-phytate phosphorus	0.13	0.29	0.45	0.59

^1^ Provided per kilogram of diet: vitamin A, 8000IU; vitamin D3, 3600IU; vitamin E, 21IU; vitamin K3, 4.2 mg; vitamin B1, 3 mg; vitamin B2, 10.2 mg; folic acid, 0.9 mg; pantothenic acid, 15 mg; niacin, 45 mg; vitamin B6, 5.4 mg; vitamin B12, 24 μg; biotin, 0.15 mg; copper, 6.8 mg; iron, 66.0 mg; zinc, 83.0 mg; manganese, 80.0 mg; iodine, 1.0 mg; Se 0.3 mg. ^2^ All nutrient levels were calculated, except values of calcium and total phosphorus were measured values. ^3^ L = group contained 0.13% nPP; M = group contained 0.29% nPP; N = group contained 0.45% nPP; H = group contained 0.59% nPP during 1–8 weeks of age.

**Table 2:** Composition and nutrient levels of 9–26 weeks diets (%, air-drying basis).

Items	9–12 Week		13–16 Week		17–20 Week		21–26 Week
	n ^3^	h ^3^	n ^3^	h ^3^	n ^3^	h ^3^	
Ingredients (%)							
Corn	67.23	67.13	68.84	68.84	69.65	69.65	65.05
Soybean meal	23.50	23.50	19.90	19.90	22.80	22.80	24.2
Wheat bran	5.60	5.60	7.0	7.00	—	—	—
Dicalcium phosphate	1.50	1.90	1.20	1.55	1.70	2.10	1.70
Zeolite powder	—	—	1.00	0.85	1.00	0.85	—
Limestone	1.20	0.90	1.20	1.00	4.00	3.75	8.2
Salt	0.30	0.30	0.30	0.30	0.30	0.30	0.30
Choline chloride	0.05	0.05	0.05	0.05	0.10	0.10	0.10
L-Lysine HCl	0.07	0.07	—	—	—	—	0.33
DL-methionine	0.16	0.16	0.12	0.12	0.12	0.12	0.12
Threonine	0.05	0.05	0.05	0.05	—	—	—
Premix ^1^	0.34	0.34	0.34	0.34	0.33	0.33	0.33
Nutrient levels ^2^							
Metabolizable energy (MJ/kg)	11.72	11.72	11.72	11.72	11.30	11.30	11.26
Crude protein	16.51	16.51	15.51	15.51	15.52	15.52	16.04
Utilizable methionine	0.40	0.40	0.35	0.35	0.37	0.37	0.38
Utilizable methionine and cystine	0.66	0.66	0.6	0.6	0.64	0.64	0.65
Utilizable Lysine	0.85	0.85	0.75	0.75	0.75	0.75	0.78
Utilizable Tryptophan	0.18	0.18	0.17	0.17	—	—	0.16
Utilizable Threonine	0.66	0.66	0.61	0.61	—	—	0.59
Calcium	0.95	0.89	1.06	1.02	2.51	2.53	3.60
Total phosphorus	0.62	0.67	0.58	0.63	0.61	0.66	0.65
Non-phytate phosphorus	0.39	0.45	0.34	0.39	0.39	0.45	0.39

^1^ Provided per kilogram of diet: vitamin A, 8000IU; vitamin D3, 3600IU; vitamin E, 21IU; vitamin K3, 4.2 mg; vitamin B1, 3 mg; vitamin B2, 10.2 mg; folic acid, 0.9 mg; pantothenic acid, 15 mg; niacin, 45 mg; vitamin B6, 5.4 mg; vitamin B12, 24 μg; biotin, 0.15 mg; copper, 6.8 mg; iron, 66.0 mg; zinc, 83.0 mg; manganese, 80 mg; iodine, 1.0 mg; Se 0.3 mg. ^2^ All nutrient levels were calculated, except values of calcium and total phosphorus were measured values. ^3^ n = group contained 0.39% nPP; h = group contained 0.45% nPP during 9–20 weeks of age.

## Data Availability

The data presented in this study are available on request from the corresponding author.

## Supplementary Materials

The following are available online at https://www.mdpi.com/article/10.3390/ani11123546/s1, Table S1: Optimal added non-phytate phosphorus level of layers during brooding period as calculated based on broken-line models.
