# Supplementary material for: Phosphorus Restriction in Brooding Stage Has Continuous Effects on Growth Performance and Early Laying Performance of Layers
Source: Animals (Basel). 2021 Dec 14;11(12):3546. doi: 10.3390/ani11123546 (PMC8698199; doi:10.3390/ani11123546)
Supplement: Supplementary file 1 [file animals-11-03546-s001.zip › animals-1462400-supplementary.pdf]

Table S1 Optimal added non-phytate phosphorus level of layers during brooding period as calculated based on broken-line models

Dependent variable	Regression equation	Coefficient of determination (R2)	Optimal added non-phytate phosphorus level %	P-value
BW	y=321.87+847.9x (x<0.32)<br>y=321.87 (x≥0.32)	0.9184	0.32	<0.0001
ADG	y=4.975+16.1x (x<0.32)<br>y=4.975 (x≥0.32)	0.9301	0.32	<0.0001
ADFI	y=15.17+36.72x (x<0.31)<br>y=15.17 (x≥0.31)	0.8536	0.31	<0.0001
Tibial length	y=66.79+55.25x(x<0.32)<br>y=66.79 (x≥0.32)	0.9352	0.32	<0.0001
Daily tibial increment	y=0.70+0.94x (x<0.33)<br>y=0.70 (x≥0.33)	0.9374	0.33	<0.0001
P content in tibia	y=0.04+0.13x(x<0.32)<br>y=0.04 (x≥0.32)	0.7524	0.32	<0.0001

BW= body weight, ADG= average daily gain; ADFI= average daily feed intake
